# Odontological Society of Pennsylvania—Tenth Anniversary Meeting

**Published:** 1889-04

**Authors:** 


### ODONTOLOGICAL SOCIETY OF PENNSYLVANIA.

### TENTH ANNIVERSARY MEETING CONTINUED.

**Discussion on Dr. Truman's Paper on The Pulp, and Treatment of Pulp Canals.**

*Dr. S. G. Perry, New York City*—There is little I can say except to commend the paper in every particular. From microscopic observations of the contents of pulp canals, I find that when first opened, if there is a disagreeable odor, there is always a very lively condition microscopically. As such teeth are the ones from which trouble may arise upon disturbance, it does not seem too much to assume that microscopic life may be directly or indirectly the cause. Whether such odors are due to the rapid multiplication of germs or to chemical action resulting from such multiplication, is hard to say; but it tallies with clinical experience to expect disturbance when such teeth are opened if the most prompt and active antiseptic measures are not resorted to. It does not seem right to me to attribute the trouble to the contents of the pulp canal alone. We must look further, for, as Dr. Truman says, there is a large proportion of once living matter in the tubuli themselves, and to fill the pulp canal before such animal matter has been sterilized seems to me unwise treatment.

As to the removal of the pulps of teeth that have never been inflamed—if they are distroyed at all—I think there can be no doubt, on general principles, that it is well, if there is a healthy condition, to fill the canal as quickly as possible. If it is left open particles of food, mucus, etc., get in—substances that will produce the very trouble the pulp itself will produce if not removed; so it is best to close the teeth as quickly as possible.

The best method of filling the roots of teeth seems to be an open question. For many years I have been in the habit of using the gutta points. I believe they originated in my office. I use them as much as any other means, because it is possible to carry thin points to the end of the roots, and by the solvent action of chloroform get a perfect fit to the walls of the canal, as well as to be more certain of closing up the open mouths of the tubuli. My objection to oxychloride is that the air becomes confined before the instrument, and it is very difficult to get the filling to the end of the root. This will be easily shown by the attempt to fill the roots of teeth out of the mouth, or better still, the closed end of glass tubes drawn down to resemble the shapes and sizes of the pulp canals of teeth. In filling the canals of teeth there must be no open spaces left, as they become receptacles, and being filled by infiltration become as great a source of danger as an unremoved pulp.

And yet I consider that there is, after all, an objection to gutta-percha. It is in the fact that it is somewhat absorbent and that it is not antiseptic. Sometimes when removed, after many years, there will be a disagreeable odor from it. The filling of a pulp canal should be as indestructible and non-porous as possible. I question therefore if it is not true, as claimed by Dr. Jack in the "American System of Dentistry," that the very small part of the pulp canal near the end of the root can be filled as accurately with gold as by any other known means.

I have in the last few years many times filled the tips of roots with gold in the manner described by Dr. Jack, and always with the feeling that I had made a close tight filling, free from pits and holes. After reaching the large part of the canal I see no advantage in using gold. In other words, when I can be perfectly certain of filling with oxychloride of zinc without danger of air bubbles, I know of no better substance. I suppose I fill the tips of the canals about as often with gold as with gutta-percha, but the larger parts of the canals I almost always fill with oxychloride of zinc. Drying the tooth as much as possible I saturate the root with chloride of zinc in the hope of sterilizing the tubuli, and then fill the canal with the oxychloride of zinc. These methods are applicable to good healthful teeth. The roots of teeth that have been diseased I often fill in such a manner that the root filling can be removed without too much trouble. For such cases I often use tapered gold wire filed to fit the root fairly, and reaching if possible to the apex. This I plunge through the oxychloride or the solution of gutta-percha, as the case may be. The large end of the wire I notch or bend over in the form of a ring to facilitate removal in case of need. Although I do not put in fillings expecting they will be removed, yet it is comfortable to know that in case of need it can be rather easily done.

*Dr. Joseph Head, Philadelphia*—I do not come up here because I want to speak, but because I have something to say. I stand here as a person who fills roots with cotton, and I wish to explain my reasons for so doing. If I am wrong I will gladly change my course, but at present my sole desire is to see both sides of the question represented.

It has been justly said by the last speaker that oxychloride of zinc, phosphate of zinc and gutta-percha are porous ; that taking up the fluids of the blood they become saturated with putrefactive matter. Gold has been lauded as a root canal filling, and when the apex of the canal can be readily reached, gold certainly can be legitimately used, as it makes a thoroughly tight joint. But it is universally known that the roots of molars usually are flat and tortuous, that the accurate adaptation of either gold or gutta-percha is impossible. And when the utmost care has been used, and even when the canal has been thoroughly filled, the risk of alveolar abscess is always present ? There always is a strong probability that the outer portion of the apical foramen is unfilled ?

If, therefore, recognizing these facts, a dentist fills a canal with an irremovable filling, should trouble arise, and the patient return, the uncomplimentary remarks of the patient will have some degree of justice.

The dentist is in a bad scrape ; he does not know what to do ; the tooth is jumping ; the patient is in anguish and wishes to be relieved, and the gas is imprisoned at the apex of the root, and the unfortunate practitioner seizes his engine and proceeds to vent the aching tooth. He has the unpleasant task of perspiring for two or three hours, but what is worse he almost kills his patient.

How much better would it have been both for him and his victim, if instead of the irremovable filling he had inserted a cotton dressing, soaked in carbolic acid. Had the cotton been used, two minutes work with a spear pointed drill would have given the patient almost instant relief.

*Dr. L. Ashley Faught, Philadelphia*—I have in my practice seldom filled a root with anything other than cotton, and, if my experience with it is as favorable in the future as it has been in the past, I shall continue to follow the same line of practice. Dr. Head has presented to you the reasons why he likes to use cotton fillings, and I agree with every sentence.

There is a little word of caution, however, which might be well mentioned in this relationship—that it is important to be careful in choosing with what you saturate your cotton. There are drugs which certainly seem to disintegrate the fibre of the cotton, and if you should experiment first with one thing and then another, be careful, for upon removal you may find the cotton broken up and in such a shreaded condition that it will be worse than boring out a gold filling. It is almost impossible to get at the apex.

My choice of dressing is the one suggested by Dr. Kirk: Iodoform disguised with oil of cinnamon. It seems to be the most satisfactory thing for a final filling; and when the cotton is packed thoroughly and tightly into the root, it is as solid and impervious as anything can be.

*Dr. Geo. S. Allan, New York City*—I like a bold man very much, and therefore I am very much pleased at the stand taken by the two gentlemen who last spoke to you in favor of that much abused cotton filling. I have used it for years in certain cases, and my success with it has been as favorable as with any other material, though I have used gold largely; in fact, uniformly use it where a root can be readily filled or gotten at, cotton saturated in some strong antiseptic, such as oil of cloves, or better still, tyron, can be packed more thoroughly into a fine root canal, filling it up more perfectly than any other material that I know of; and I have taken out cotton that has been saturated in tyron, packed into a root for 15 months, and it was just as sweet as the day it was put in, an an almost imperceptible odor of tyron could be distinguished.

It behooves us to be very careful in condemning our material in use as root filling, owing to popular prejudice. There is a prejudice against cotton, and it is talked very slightingly of. Pure cotton, if packed into canals, and then excluded from the moisture of the mouth, makes as perfect a filling, I may say, as gutta-percha. It is not as porous, and therefore is not as subject to the changes that gutta-percha is liable to. One of the best methods of preparing cotton for root filling purposes is as follows: Soak it for some time, beforehand, in one per cent. solution of mercuric bichloride. Then dry it carefully. Then before using it wrap it carefully around the proper broach, taking pains to employ only cotton enough to fill the canal, and give it a solution of resin in ether, dry off with bibulous paper the excess of resin, and then pack it into the canal. Cotton so prepared is almost indestructible. Bacteria cannot obtain a footing in it or grow in its meshes. It is taken for granted that all root canals are sterilized before filling, no matter what the nature of the filling material employed. I advocate cotton only as one of many useful materials available for the purpose.

*Dr. J. N. Crouse, Chicago*—I am a little disappointed in coming back to this old city, where I received the first part of my dental education, and listening to-day to my old teacher, to find him advocating about the same practice and holding the same views that he did twenty-five years ago, not having advanced any in this one direction. I cannot agree with him in his views on capping pulps, for if I understand his meaning correctly he condemns almost in toto pulp capping. What he said on the subject was little, but enough to show that this was his view. I stand here an advocate of pulp capping and claim a successful practice in this line. If the gentleman is not able to cap pulps, I am and successfully, and consider the destroying of pulps and filling of roots the last thing to do. I formerly filled roots with gutta-percha and gold, using a little gutta-percha in end of root, which does not prevent me from forcing the gold as far as it will go, the gutta-percha occupying the space which would otherwise be left vacant, unless some plastic was used.

I should cap the pulps of all teeth when the dentine is sensitive; that is when it responds to scraping of excavator and gives off sensation, which I believe to be one of the best tests of a healthy pulp. I should apply carbolic acid freely getting a complete coat of coagulated albumen over the point of exposure, cap with oxychloride of zinc, being very careful to apply the capping in a skilful manner, believing it to be one of the most delicate operations in dentistry, and being sure to have a good solid base for the cap to rest upon, having removed sufficient of the softened portion to make sure that the cap rests solid, and filling over this with whatever material seems best for the permanent filling. As these were my views when I came here, I do not expect to go home and apply arsenic or any other destructive agent to pulps, but shall continue to cap them, having great faith from a long and successful experience in this line of practice.

I do not wish to be understood as claiming that all pulps that are capped remain alive; a certain per cent. of them die, giving no trouble to the patient, but remaining entirely comfortable and the teeth not discoloring; and only by applying an extreme of cold and heat would you know the pulp was not alive.

Another per cent. will give trouble, and the patients come back to you, or to some one else, after capping, and will receive the treatment as laid down on the paper, which I consider sound both as to manner of getting ready and the filling, except perhaps the use of oxychloride instead of gutta-percha; but I think I would as soon risk one as the other.

There is one thing that is lost sight of in deciding between capping or destroying pulps, and that is that quite a per cent. of teeth, even when the roots are filled as well as can be, frequently give trouble.

I did not expect to come here and hear of so many successful operations by filling the roots of teeth with cotton. I thought that practice had died out long ago. This cotton practice has a redeeming quality spoken of by one of its strong advocates here to-day, which is, by leaving the ends sticking out, it can be removed quickly when the tooth gives trouble; a very wise precaution which will be often needed.

*Dr. C. E. Francis, New York City*—I listened to Prof. Truman's paper with much interest; it contains a great deal of sound sense. The subject is an old one, but at all times important and interesting. Dr. Turman said so little regarding the capping of pulps that I did not suppose his subject covered this mode of procedure. I imagine that but few of us here present think of devitalizing a freshly exposed pulp. I have for many years been in the habit of capping pulps when newly exposed, and have written several papers on the subject giving my reasons for so doing. As regards the matter of devitalizing freshly exposed pulps, I believe the time has passed for this sort of practice. Prof. Turman, however, referred to " sick pulps " or pulps in a pathological condition, and I fully agree with him in his treatment of such cases, for I have but little faith in the restoration or salvation of pulps that are sickly or in a partially congested conditon.

As regards the material for filling root canals, I have many times heard it argued that it made little or no difference what substance was employed, if only the canals were properly prepared before receiving the filling; and it seems to me that this is about the case. Gold, tin, gutta-percha, wood, cotton and the zinc stoppings have all been used with success. Cases have come to my notice where roots filled with cotton have remained in good condition for many years. A prominent and excellent dentist of New York has used cotton as a root fillng in very many instances, and a number of teeth so treated have come to my hands. I had occasion to remove the fillings of several of these teeth, and found the cotton thoroughly packed and apparently as clean and odorless as when introduced. Packed in this manner the cotton would not be apt to absorb moisture from the foramen at the end of the root. Success in root fillings is in great part due to the preparation of the canals, much more so then in the choice of materials used. They should be thoroughly cleansed of all debris, washed well with tepid water; disenfectants, or antiseptics injected, and made perfectly dry with warm air. I never boast of my ability and in such cases am not sure of perfect results. But as regards freshly exposed pulps, I would always endeavor to save their vitality, and if successful but once in three cases it is worth our effort to try. In the majority of cases, however, where the pulps are healthy I believe they can be saved.

*Dr. W. A. Atkinson, New York City*—I never allow my pupils to kill pulps except where they cannot control the case, as in persons who want to travel, and where pulp stones must be removed to secure usefulness of the root.

As to the paper the doctor said squarely that when pulps came to him exposed, he let other people take care of them. The time is upon us when pathological chemistry must be accurately studied before we can grasp the situation and do our whole duty. We know that chloride of zinc and oxide of zinc mixed together produce a hard body, and whenever any body is crystalized with a great number of crystals, there must be spaces between. It is the zinc that acts upon the fibrils forming a "hydrochloride of albumen" in the root. When the root is well filled with any fit substance, it should never be removed. Because if you have a tooth filled to or even a little beyond the foramen, the end becomes encysted, and there is no occasion to remove it. There is a great deal that goes to make up success that is not scientific; though it is, at the same time, the very highest science occulted.

Now, with regard to pus and the process, we heard to-day that it was the leucocytes under the inflammatory process, escaping into the cavity of the abscess that become pus corpuscles. If alive until next June, I hope to be able to put this matter straight, as I am preparing a paper on the origin of pus. It is impossible for leucocytes to pass the hardened congested tissue of an abscess in sufficient quantity to supply the pus to fill it. A retrograde molecular metamorphosis melting and breaking up the tissue elements is the antecedent of an abscess in every single instance. There must be living tissue and micro-organism in the neighborhood of the debilitated protoplasm before there can be any pus. Abscess is local and dependent upon a deterioration in the juices of the flesh (the substances ready to be appropriated by the process that we call nutrition). I merely name these points to show how far and endless the discussions could be if fully elaborated.

*Dr. C. E. Francis, New York City*—In my previous remarks I hope I did not give the impression that it was my custom to fill root canals with dry cotton. The idea intended to convey was that success depended more in the preparation of the canals than in the filling material. In conversation with the late Dr. Varney, many years ago, he remarked that he considered oxychloride of zinc the best stopping for roots. He believed it possessed antiseptic properties and would remain permanently. There is some difficulty in introducing this material into the canals. Small fibres of cotton wound around a broach answers a good purpose for carrying the zinc paste into the roots.

*Dr. W. H. Dwinelle, New York City*—Dr. Perry made the remark that I had had an experience which antedated the experience of many on the subject of filling root canals, and I was about to say that also with reference to the capping of nerves.

I think it was in 1842 or 1843, I was constrained to advocate the retaining of the vitality of the nerves of the teeth in cases of deep seated caries. It was customary with many of us, and with the profession generally, at that time to excavate a cavity until all the decay was taken out, and when the decay was deep and the dentine largely disintegrated, it involved the uncovering of the nerve; and according to the orthodoxy of the day, a nerve exposed must necessarily be destroyed ; at that time I advocated the retaining the vitality of the nerve leaving this partially decomposed bone as a capping for it. I can show you to-day evidence to persuade you there were instances wherein this partially decomposed bone, allowed to remain, has been recalcified. It is the office of the nerve to deposit secondary dentine, and cover its retreat so that in extreme old age it is entirely obliterated. I was abused roundly for it, and in conjunction with the articles published on the subject, I was accused of leaving decay in the tooth, a very unpopular thing, especially as our operations are designed to save teeth from decay.

Now, in regard to exposure of the pulp, I was, perhaps, one of the earlier dentists who recommended saving the nerves of the teeth. I have been successful in a large majority of cases. I have within a few weeks examined some teeth whose nerves were exposed and capped away back in the forties, and found them entirely intact and alive and covered with a calcified capping. With regard to filling nerve canals, I think I have the advantage of the experience of those as far back as Hudson and Maynard. Their methods are familiar to you all. My method of filling nerve canals, if it is a case when it is absolutely necessary for it to be destroyed, devitalize it, remove, medicate and then fill at once. If it is an old tooth, dead and charged with decomposed matter, adopt methods to absorb it out, treat thoroughly until you are satisfied the fibriles have been thoroughly coagulated, sterilized and inert, then fill it thoroughly.

Then for years I filled with gold, though not so much now, not the entire canal. I roll my gold on a Swedish broach so that it forms a sort of attenuated cone, measured the length of the root, and then take a Swedish broach and made a slight right angle at its extremity. It is then made to slip down until it has passed the foramen. This can readily be appreciated by the delicate hand of the manipulator. It is then marked and drawn out, when the rest of the filling instruments are gauged by it.

It was my custom to fill nerve canals, until recently, entirely with gold—always with gold at the extremity I rarely have any trouble with teeth; when I do, I consider that I have not done my work thoroughly; that I have not allowed sufficient time between my treatment and filling. If I have any trouble, I do not remove the filling at all, but resort to external treatment with the drill. I do not think it is extremely painful. I simply put a drop of cocaine on the gum over the extremity of the root, and with a drill suddenly open, through the process, to the extremity of the root, and there I accomplish all that I could by taking the filling out. I rarely have trouble after this.

I am not in favor of the use of cotton pure and simple for filling nerve canals; if you use it in conjunction with resin, wax or oxychloride of zinc, the virtue of the operation must be attributed to these materials, and not to the cotton, which only facilitates their introduction. More depends oftentimes upon the intelligence, skill and delicate manipulation of the operator, than anything else; but you cannot hermetically seal a cavity with cotton alone.

*Dr. James Truman, Philadelphia*—I think, in justice to myself, and in response to my friend, Dr. Crouse, of Chicago, who says I have not learned anything in twenty-five years, that I should say something. I believe I have acquired something in that period and have, I am sure, passed through all the stages of capping, and have tried all recognized modes of treatment. He will, however, permit me to say that capping was not the subject of the paper, and was referred to only incidentally among others as the basis of my argument. But as this subject has been brought into the discussion, I wish to repeat that there are conditions where pulps may be saved by this process, and also pathological states where it is impossible to save them. I should be untrue to my convictions did I not recognize the fact of the possibility of saving pulps after continued exposure and irritation.

The point that I wished to make in the paper was not antagonism to cotton filling any more than to capping. While I oppose both as a general practice, there may be times when both may be a success. The reason given for its use, that is easily removed, seems to me a fallacious one. As Dr. Atkinson truly says, why do we wish to remove a canal filling that was put in to stay? I never remember a case where this seemed to be required that external treatment did not answer equally as well. A gold filling, to be of any value, must be packed in as solid as the mallet can make it. Nothing is, therefore, to be gained by attempting its removal. I do not want a filling that can be withdrawn. To make it of this character implies a doubt and suggests a possibility.

That which I wished to call especial attention to, in the paper, was the condition of the tubuli of the teeth. The organic matter contained therein when left without treatment is followed, eventually, by discoloration of the tooth. I endeavored to impress upon you the necessity of placing something in the canal that will act upon the dead tissue in the tubes, and I suggested for this purpose chloride of zinc in solution. The well known property of this agent, as a coagulator, makes it an especially desirable agent in this connection. Its affinity for moisture will cause it to penetrate deeply, and where coagulation is effected decomposition is impossible. This opinion is based upon clinical experience and to some extent upon microscopical observations. I have tested it so long that I feel every confidence in it, and do not pretend to fill a tooth before I have had the canal under the effect of this agent for several days, as the use of chloride of zinc for this purpose is original and of necessity will need confirmation from others, I desired it should not be overlooked in the discussion.

In regard to the remark of Dr. Atkinson that the leucocytes, found in an inflamed pulp, where the product of metamorphoses, I have this to say, that I do not think we know enough in regard to it to formulate an answer. My inference from observation is that they are not present through retrograde metamorphoses, but the result of migration.

Subject passed.

DISCUSSION ON DR. WATER'S PAPER ON REMOVABLE BRIDGE-WORK.

*Dr. W. D. Dwinelle, New York City*—This is a new field for dentists. There are objections to the old system of bridge-work. Cleanliness is often interfered with very materially. If the piece of work gives way, or breaks in any particular, or a porcelain crown breaks off, it is not possible to repair it permanently unless you destroy the whole device and commence *de novo;* but by the system of removable bridges we are able to take it out at leisure, correct and renew it, and put it back to its place. Furthermore, in the matter of cleanliness, this new system of Dr. Water's removes the objection very materially. I am pleased with his system of telescoping, so to speak, his bridge-work on to other teeth, preparing the crowns by cementing a gold cap upon the natural crown, and when taking an impression of that and fitting a cap which is made to slip upon the cap-crown already in the mouth. Then the split-post, in conjunction with it, gives it firmness, which is very desirable. We are not obliged to resort to the old system of cementing bridge-caps on permanently, which is objectionable.

Dr. Parr has invented a system of removable bridge-work of a very ingenious and practical character, doing away with most of the objections to work of this kind.

I regret that he is not present to exhibit to us his new system. I believe, however, he has held a clinic during our session illustrating his method. He regards his invention as a fixture, permanent when in position, and easily removed and replaced at the will of the patient.

I am very much interested in bridge-work. I claim that in a treatise published in 1855 in the old "American Journal of Dental Science." I there published to the world, the system of cap-crowns and so-called bridge-work, which was my invention.

*Dr. A. G. Bennett, of Philadelphia*—I am somewhat known as an advocate of bridge-work, but have no desire to be known as such except in so far as this mode of substitution conforms to dental science and is constructed on correct mechanical principles. I think the progress thus far made is as rapid and satisfactory as could be expected.

The idea of cleanliness has been very properly more emphasized than any other. With the removable bridge we get rid of several objections that were formerly inherent in certain kinds of cases; cleanliness is secured and repair made easy. With the first method —that of cementing in securely—I cannot say that I have had any trouble that could be charged to the system itself; but in cases of chronic trouble about the roots or low recuperative power, the improved system has several advantages.

With the first method the bridge and the abutment caps were one piece; now they are separate. There are two kinds of removable bridges: the one to be removed by the operator, the other by the patient. If the abutment or anchorage teeth are properly prepared and entirely encased in gold caps, we have done our utmost permanently to protect and preserve the foundation of our work. Of course, leaks and ledges and irritants must be carefully provided against in each case. Bridges designed to be removed by the patient are usually retained by a spring. There seems to me to be a weak point in this method in that the spring may be loosened or unsprung, and then the bridge is open to the same objections that many small partial plates are—it may get into the throat. I have not tried the spring method, but have constructed some on a modification of this principle, and now make all my bridges so that they can be readily removed by softening the gutta-percha which holds them in place.

But I will first speak of a method of my own. About a year ago I had some cases that required that the gold should be kept entirely out of sight. I did this by encasing the inner half of the teeth—molars and bicuspids—with half caps that were anchored in a groove that passed between the cusps and down the proximal surfaces beneath the gum. I used the Bing bridge teeth which showed only a mere line of gold between, and the end which rests on the gum, the denture being anchored with gutta-percha. These teeth are prepared by grinding off the rounding part of the palatal surface, which is afterwards restored with gold solder.

As to bridges resting on the gum, I thought at first that this was not a good plan; but I find, by examining cases set in this way and by a little experience with the method, that, if the porcelain fits accurately and is brought to bear lightly against the gum, there is neither irritation nor uncleanliness. This resting on the gum is one of the chief points in the Brown bridge, which is all porcelain baked around a platinum skeleton.

One of the worst objections to the outward sloping palatal or lingual surfaces of the gold cusp teeth is that they are not exposed to attrition, and cannot be kept polished. Under such conditions, a solder surface will tarnish sooner or later, and then food debris becomes adherent, and the parts are uncleanly. The Bing teeth, as I use them, must, of course, have a solder palatal or lingual surface; but since this is perpendicular, it can be readily reached by the tongue and brush.

In other cases, as already stated, I have used a modification of Dr. Parr's system. This system consists essentially of a socket in the abutment-cap and a tongue containing a spring on the bridge proper. The socket on the tooth-cap and the pin on the bridge are roughly illustrated by the pin and aperture on a molding flask. The tongues are pressed into their respective sockets, and the denture is then cemented into position, which sets the caps parallel, allowing the part that spans the space to be readily removed. I have modified this method by leaving out the spring and anchoring the bridge with gutta-percha. I make the socket round, and fit the pins a little loosely, to leave a space for a film of gutta-percha, the advantage being that no spaces are left and all moisture excluded. I do not wish to be understood as condemning the spring system, which can be made very strong and durable; but, for the reasons given above, I prefer the modification which I have mentioned.

I will add that I consider bridge-work to have passed beyond the stage of controversy. It is based on principles as well established as those of any other system of substitution, and a method of inserting crowns without roots does span the chasm which has heretofore existed between crowns and plates. Each one has its proper place, and all are needed to meet the varying conditions. A plate is a foreign body. A bridge is the same, with this difference: that the latter, when properly made, is not half so injurious as the former. Defects have been, and are still being eliminated, and objectors are changing their attitude or subsiding into silence. We are making progress, and have every reason to believe that we have contributed something to dentistry that is of permanent value; and I will give it as my final opinion that bridge-work has come to stay.

*Dr. W. H. Dwinelle, New York City*—I have a personal interest in this matter, as I introduced the system many years ago; and in order to justify myself in what I have said on this occasion, I beg to refer those present, who have the opportunity of looking into the back volumes of the "Cosmos," to an article by Dr. Stover Howe, on The "Evolutions of the Tooth Cap, Crown and Bridge-work,"

in which he justifies me in all I said this afternoon. He referred to my original article published in the old "American Journal of Dental Science" in 1855.

I shall esteem it a personal favor if you will refer to this article by Dr. Howe, I think you will all justify me in what I have said. I did not know Dr. Howe at the time, so there could have been no collusion in reference to the matter. It was published in the "Cosmos," June, 1886 ; so it is not far back.

*Dr. W. A. Atkinson, New York City*—I am delighted with the exhibit before this body. The roots of teeth are inserted so that their crowns may best receive the occlusion of the opposing teeth. The pericementum is a nice, little cushion to break the immediate force of the occluding energy in mastication, especially in biting off hard substances.

If on over shock this pericementum, under the law of nutrition, its action will melt the lime salt, so that without any inflammation that is recognizable, that tooth will become so loose that you may think you can pull it out with the fingers, but you will not be able to do so, as the softened tissues will hold it.

Takes these two teeth (pointing to the specimens), the lime salts are simply melted in situ, and when the quietness that is necessary for the consolidation was secured they became firm in condition. Place the tooth in the position it is desired to have it, and give it steadiness enough to allow hardening of the lime salts of the osseous structure of the matrix, and you will have that in a healthy condition, and be able to bear all the use required of it.

I have seen cases from the Sheffield house of the International Tooth Crown Co., with bridges attached to and carried over loose teeth, and roots which were very unsatisfactory to the patient, discharging pus for months, which under proper treatment became healthy, firm, and useful, some without resetting, and others involving resetting of the pieces, and still others wasted beyond recovery.

Subject passed. To be continued.

### EXHIBITS AND CLINICS AT ANNIVERSARY MEETING OF THE ODONTOLOGICAL SOCIETY OF PENNSYLVANIA.

The S. S. White Dental Mfg. Co. in their exhibit made a specialty of electricity as applied to dentistry, the electric current supplied from street wires or otherwise. An Electric Dental Engine, and a device to stop motor instantly at will; Rotary Fan; To and fro Fan; Electric Plugger; Hot Air Syringe; small electric lamps of from 16 to $\frac{1}{2}$ candle power for use in the mouth; Laryngoscope, Cautery, etc., were shown. They also exhibited a slip joint and duplex attachment to engine; Bonwill's mechanical mallet; also a new line of dental instruments.

Mr. Gideon Sibley exhibited his felt gold, which is claimed to meet a long "felt" want in the profession. Also a needle foil carrier for same, together with a general line of dental supplies.

R. S. Williams, of New York, displayed a fine assortment of gold foils, gold plate, cylinders, pellets, and blocks, including his specialty "Crystalloid Plastic Gold," which has many friends in the profession. It was freely tested during the exhibit, and seemed to be all that was claimed for it.

C. Ash & Son, of New York and England, exhibited a full line of their celebrated teeth, as well as general dental supplies.

The Wilmington Dental Manufacturing Company made a display of "Wilmington Teeth," including Dr. Genese's Pinless Teeth and specialties, Wardwell's treadles, a new cabinet, and the new Register Engine, Hand Piece and Mallet, a description of which will be found in Domestic Correspondence Department.

The Keller Medicine Co., of Ft. Wayne, Indiana, showed a large assortment of non-secret medicines, together with new dental chairs, motor, gas apparatus and dental materials.

Seabury & Johnson, of New York, displayed their Hydronaphthol Specialties, Antiseptic Cotton, Napkins, etc.

Henry C. Blair's Sons, Philadelphia, exhibited a full line of Tooth Powders and Washes.

Parke, Davis & Co., of Detroit, were represented in their various preparations of Cocaine, and Antiseptic Tablets, as well as a preparation or Powdered Bone, which they recommend as a true nerve and bone food.

Waltham Emery Wheel Co. exhibited a large line of Dental Wheels and points for dry grinding.

Davidson Rubber Co., of Boston, displayed their specialty of "Velvet Rubber Dams," as well as all forms of Dental Dams.

H. K. Mulford & Co. showed an extensive list of Dentifrices, Powders, Antiseptic Tablets, Capsicum Pads, including their Silica Fluoride of Sodium preparation for a dentifrice.

Edward Rowan, of New York, exhibited his preparation of gold, and specialties in filling materials.

H. D. Justi, of Philadelphia, displayed teeth and dental supplies.

Dr. Seabury, of Providence, R. I., made an exhibit of the Seabury Vulcanizer.

W. M. Speakman showed, besides several novelties, a general supply of dental goods and teeth. The Parson's electric hot air apparatus attracted considerable attention.

W. P. Green exhibited Engine Burrs.

Dr. W. G. A. Bonwill demonstrated the use of hard rubber corundum disks for sharpening burrs.

Dr. J. W. Ivory, Philadelphia, displayed his Improved Rubber Dam Clamp, including his napkin clamp, which seems to embrace many desirable qualities.

*The International Dental Journal* distributed sample copies and took subscriptions.

Dr. C. C. Carroll, of New York, exhibited his automatic gas furnace, flask, and pneumatic crucible for casting aluminum dentures, bridges, and crowns.

CLINICS.

Dr. H. C. Register, of Philadelphia, gave a clinic, contouring a right superior central incisor and building down a left superior central incisor with cylinders (finishing with foil) with the Register Mechanical Mallet and compensating engine.

Dr. T. S. Waters, of Baltimore, exhibited three pieces of removable bridge work in the mouth. The first was a gold crown sliding over a cap attached permanently to the teeth or natural crown. This cap has on one side a longitudinal groove. The gold crown has soldered on the inside a spring catch, which works in the groove on the cap, and holds the crown permanently in its place. The second device was that of the "Box-Cap and Split-Post"—a box-cap being fitted permanently to the root, and the split-post being soldered to the plate having the teeth. The doctor also showed a third device—that of soldering to the side of the gold crown covering the natural teeth a split pin or post, which is inserted into the teeth attached to the denture.

Dr. Waters, in addition, exhibited an entire denture supported by six anterior teeth; as well as a full denture supported by two cuspids, a bicuspid and a molar. All these pieces are under perfect control of the patient, who can take them out at will, and can thus be kept in a perfect sanitary or hygienic condition.

Dr. A. G. Bennett, of Philadelphia, exhibited a "Rapid Excavator," for large cavities; also a plugger—two points in one shank, for the hand or automatic mallet.

Dr. H. A. Parr, of New York City, showed a removable bridge carrying a sixth year molar and two bicuspids. The attachment was made by a spring fitting into a slot made in the cap on the twelfth year molar. The plate rested on the gum, fitted very nicely, and seemed all that could be desired. Dr. Parr also exhibited his universal separator.

Prof. R. B. Winder, of Baltimore, exhibited three removable bridges—two lower and one upper attached to adjoining teeth with set screws. The doctor also showed an adjustable rubber dam clamp, which is certainly a handy, little article.

Dr. J. A. Woodward exhibited a novel and excellent appliance in the shape of an illuminating apparatus for dark days, by which the most intricate filling can be clearly seen, and with a mouth mirror the light can be reflected to see any dental approximate surface. It consists of a Mackenzie light concentrator No. 2 for illuminating gas, with reflecting mirror, and is mounted on a heavy, upright rod attached to an arm which swings from the base of the Wilkerson or High-Low Base chair. When adjusted for an operation it will follow the movements of the chair.

Dr. Woodward stated he decidedly preferred this light to poor natural light; but that the electric light shown by the S. S. White Dental Mfg. Co. was more convenient, as it could be placed further away from the work and yet give equal intensity. It also weighs less, is free from heat, and does not require the flexible rubber tubing.

Dr. J. Gardner Morey, of New York, gave practical illustrations of the methods of using his nerve and crown drills; and also exhibited his separator, with which he obtained a space $\frac{3}{64}$ of an inch in $2\frac{1}{2}$ minutes, with little or no pain or inconvenience.

Drs. W. S. How and R. Walter Starr gave a clince on Dental Inlays with porcelain.

